# Predictors for Actual COVID-19 Vaccine Uptake and Intended Booster Dosage among Medical Students of an Osteopathic Medical School in New York

**DOI:** 10.3390/epidemiologia2040038

**Published:** 2021-11-20

**Authors:** Taysir Al Janabi, Maria Pino

**Affiliations:** New York Institute of Technology College of Osteopathic Medicine (NYITCOM), Glen Head, NY 11545, USA; mpino@nyit.edu

**Keywords:** COVID-19, COVID-19 vaccine, COVID-19 booster, osteopathic medical students, medical students, vaccination, pandemic, vaccine uptake

## Abstract

Exploring future physicians’ attitudes toward vaccination is crucial as physicians’ recommendation is the top predictor for individuals to receive vaccines. This study explored the uptake of COVID-19 vaccines and the intention for future booster dose uptake among students at the New York Institute of Technology College of Osteopathic Medicine (NYITCOM). Predictors for actual vaccine and intended booster uptake were also examined. An electronic survey was distributed to Osteopathic Medical Students (OMS I-IV) in the Spring of 2021. A total of 1331 students received the survey, with 316 responses received (24%). In total, 95.3% (301/316) of the respondents reported that they already received vaccines, while 3.1% (13/316) reported that they had not yet received a vaccine. Moreover, 88.9% of the respondents (281/316) were in favor of a booster dose, which was a strong predictor for actual vaccine uptake. We identified that the Asian race, pharmaceutical mistrust, building immunity via vaccines, adequate vaccine testing, and willingness to get non-U.S. manufactured vaccines are the most significant predictors for willingness to accept a booster dose. A very high COVID-19 vaccine uptake among NYITCOM OMS was found in our study. The study also observed a high acceptance of an additional dose of the COVID-19 vaccine in the future.

## 1. Introduction

In December 2019, a case of respiratory illness appeared in Wuhan, China. Later that month, the Chinese health authorities diagnosed the person with a novel coronavirus, which became known as COVID-19, originating from SARS-CoV-2. Its spread was more aggressive than other known viral pathogens [1]. Within a few months, SARS-CoV-2 was circulating the world, resulting in 118,000 cases and over 4000 deaths by 11 March 2020, when the World Health Organization (WHO) declared the COVID-19 pandemic [1].

While the world relied on public health measures to mitigate the pandemic’s impact [2], scientists have risen to the challenge of ending the pandemic by developing a vaccine. Vaccination is one of the public health measures that has the most significant impact on reducing the mortality and morbidity associated with many infectious diseases [3]. Thus, a new COVID-19 vaccine has been viewed as the best option to save lives and restore normality [4]. By June 2021, there were 25 vaccine candidates—with different development approaches—in late clinical phases [5], five of which were backed by Operation Warp Speed (OWS) [6]: Moderna, Oxford/AstraZeneca, Johnson & Johnson/Janssen (J&J/Janssen), Merck, and Pfizer-BioNTech [4]. The United States (U.S.) regulatory authorities approved Pfizer-BioNTech and Moderna (messenger RNA vaccines) in December 2020 and the J&J/Janssen formulation (viral vector vaccine) in February 2021 [7]. These vaccines provide substantial protection against COVID-19 hospitalization [8].

Since the start of the pandemic, U.S. political leaders and pharmaceutical manufacturers have endorsed a goal of a shot for every American. This became a reality at the end of May 2021 when Pfizer, Moderna, and J&J distributed 500 million doses—enough to vaccinate all adults in the U.S [9]. While vaccines are now widely available and accessible [10], only 70% of U.S. adults have received their first dose by August 2 [11].

The lack of political will from the U.S. administration to implement a comprehensive response [12], conflicting scientific messages [13], and widespread misinformation have led to skepticism by the public about the new vaccines [14]. Initial polls during the pandemic showed that a significant portion of Americans might not choose to receive a COVID-19 vaccine when it became available [15]. The Kaiser Family Foundation (KFF) COVID-19 Vaccine Monitor showed that the share of the public who would get the vaccine increased from 63% in September to 71% in December 2020. Still, 27% of the public may not receive the vaccine even if it is proven safe, available, and free [16]. Emerging studies from different populations worldwide reported that vaccine hesitancy toward COVID-19 vaccination is high, and healthcare workers are no exception [17].

Vaccines are widely available; however, pockets of vaccine hesitancy exist. Vaccine hesitancy is defined as the delay in the acceptance or refusal of vaccinations despite the availability of the vaccines [18]. Vaccine hesitancy is a multi-factorial issue that is rarely population-specific but common in subgroups within populations. Studies conducted to assess vaccine acceptance among adults in Italy showed that acceptance levels ranged from 68.9% in Northern Italy to 91.9% in Southern and Central Italy [19]. Thus, identifying these subgroups, understanding their concerns, and investigating predictors of vaccine acceptance is imperative in developing effective and comprehensive strategies to minimize vaccine hesitancy [20,21].

While messenger RNA (mRNA) COVID-19 vaccines are effective against coronavirus and severe disease for up to six months after the second dose of the vaccines, researchers cannot confirm how long the immunity will last [22,23,24]. The chief executive officers of Pfizer and J&J announced in February and April 2021, respectively, that a booster could be needed to sustain long-lasting immunity [25]. Many studies have been exploring the duration of active immunity from COVID-19 vaccines in fully vaccinated individuals [26,27,28]. Additionally, the emergence of the different COVID-19 variants of Alpha, Delta, and Lambda, which can evade some of the body’s immune responses, may necessitate an additional vaccine dose [29]. At the time of conducting this study, a booster shot was just a thought pending more evidence to justify its benefits.

Undergraduate and medical students are a unique subgroup in their utilization of social media and their experience with COVID-19 compared to other age groups [30], making them vulnerable to misinformation and antivaccine campaigns. Research has shown that the uptake of the seasonal flu vaccine is low among medical students because of the belief that their perceived risk to the flu infection is low [31,32], putting themselves and patients at risk. A possibility of low COVID-19 vaccine acceptance among medical students might emerge because of their perceived risk.

Osteopathic medical students (OMS) are part of the healthcare force and encompass a wide variety of demographic backgrounds. Moreover, research has shown that vaccine uptake among the public might be enhanced if they receive strong recommendations from health care professionals [33]. Additionally, students can be mobilized to respond to public health emergencies [34]. Liberty University (LU) deployed its osteopathic medical students and nursing students to various places in central Virginia to enhance vaccine uptake locally [35].

With this background, this study offers an opportunity to assess COVID-19 vaccine uptake and booster dose acceptance among future physicians and identify facilitators and barriers to vaccine uptake, which can be the focus of school and health officials during vaccination campaigns. At the time of this study, a few studies were available to address COVID-19 vaccine hesitancy among medical students. Thus, this study also contributes to the limited knowledge of recognizing and addressing vaccine hesitancy in a subpopulation group whose impact is paramount in protecting and promoting at the individual and community levels.

## 2. Materials and Methods

The study protocol was approved by The Educational Research Data Committee (ERDC) and the Institutional Review Board (IRB) of New York Institute of Technology -College of Osteopathic Medicine (NYITCOM) (protocol code BHS1575 on 05/06/2021). NYITCOM has 1331 medical students enrolled in total; 50.4% of the students are female, distributed between preclinical (OMS I-II) and clinical (OMS III-IV) at two different campuses (Jonesboro, AR and Old Westbury, NY, USA). A total of 71.9% (957/1331) of the students were on the New York campus, and 28.1% (374/1331) were on the Arkansas campus. The racial/ethnic makeup of the school was 44.2% White, 38.2% Asian, 4.6% Black or African American, 2.5% from multiple races.

The research team created an anonymous electronic survey by adapting a model of determinants developed by the Strategic Advisory Group of Experts (SAGE) on vaccine hesitancy, based on a systematic review of literature and immunization manager interviews [20]. The questions were adapted from similar studies [36,37,38,39,40,41,42] to reflect on vaccine hesitancy determinants—contextual, individual and group, and vaccine-specific—identified by Larson et al. Moreover, the authors’ previous work in this area evaluated the psychometric properties of the current study survey [43]. Based on the feedback received from participants in a previous study, the survey was shortened to enhance participation, but the wording of individual questions was not changed from that study. The study’s contextual themes were health system trust, pharmaceutical industry, and knowing acquaintance with COVID-19. The study also included four individual and group influences: experience with past vaccination, health beliefs, perceived vaccine risk/benefit, and antivaccine acquaintances. Finally, three vaccine-specific influences—scientific evidence, acceptance of foreign-manufactured vaccine, and vaccination schedule—were included. The survey was distributed to all enrolled OMS email using the school’s student listservs. Responses were collected over two weeks in early June 2021, with no incentive provided to the participants for completing the survey. Participation was entirely voluntary, and the participants had the option to withdraw at any time without justifying their decision. An email reminder was sent to the students one week later.

The survey included nineteen questions, with one additional, conditional question about vaccine types if participants reported that they had received a shot. Questions were categorized into three parts: contextual influences (three items), individual and group influences (four items), and vaccine-specific issues (three items). All items were measured on a five-point Likert scale except three questions—one question about being vaccinated, another question about pharmaceutical profit, and the third about antivaccine acquaintances—were provided the option of Yes/No. Participants were also assessed on demographics (age, gender, race/ethnicity, marital status, combined household income, class year, and campus location). Campus location was included in the survey to explore if vaccine acceptance was significantly affected by the geographical location of the two campuses (urban vs. rural). Moreover, the authors wanted to ensure that the study sample was representative of the entire school population, which was distributed between two campuses. All questions had the option of Prefer not to answer. If a respondent selected “Prefer not to answer” or did not provide an answer, they were removed pairwise from any analysis using that variable but still included for analysis with the variables they responded to. The race/ethnicity question was split into two binary variables, White or other and Asian or other, for inclusion in the multiple logistic and linear regression models.

For the primary response variable of vaccine uptake, the question read, “Have you been vaccinated against the SARS-CoV-2 virus?” with yes being coded as 1 and no being coded as 2. For the primary response variable of booster, the question read, “Would you receive an additional booster dose of COVID-19 vaccine if it were required to achieve an adequate level of immunity?” with answers coded as 1 through 5 with 1 being “strongly disagree” and 5 being “strongly agree”.

We used stepwise logistic regression to identify variables with the strongest predictive validity for actual vaccine uptake and a subsequent forward stepwise regression for variables with predictive validity of willingness to take a booster vaccine. This procedure compares multiple variables simultaneously to find those with the best predictive validity while controlling for the statistical implications of multiple comparisons and removing variables with high multicollinearity from the model. The analysis was performed with SPSS 27 (IBM Corp., Armonk, NY, USA) and statistical significance was set at *p* < 0.05.

## 3. Results

The total response rate of the survey was 24%. About two-thirds (66.2%) of the participants (210/316) were from the Old Westbury campus, and 31.9% (101/316) were from the Jonesboro campus. Female respondents constituted 47.2% (149/316) of the study sample. Our study sample’s gender, racial/ethnic, and campus location characteristics were consistent with the general student population at NYITCOM. Table 1 summarizes the characteristics of the study participants at both campuses.

After performing a logistic regression for predicting vaccine uptake, only the booster variable was significant (W(1) = 12.408, *p* < 0.001). When the next most significantly related variable was entered into the model for the stepwise regression, White, it became insignificant. This means the model was reverted to the model with just booster as a predictor. This is demonstrated in Table 2 below, with the final model being shown in step 1. The odds ratio of 0.298 demonstrates that a one-unit increase in the booster variable (being more willing to get the booster) multiplies the odds of the person not receiving the COVID-19 vaccine by 0.298 (it is less likely they did not receive the vaccine). The final model’s Cox and Snell R Square value is 0.057 and the Nagelkerke Square value is 0.278.

Therefore, the best way to predict if an individual has received the vaccine already among OMS is to gauge their willingness to get another booster shot later, meaning that future willingness to get a vaccine is highly predictive of current vaccine willingness and compliance.

To break down the predictors of future vaccine willingness, a separate model was run to predict the response to the booster variable. Similarly to the logistic regression for vaccine uptake, the multiple linear regression for booster was performed with a forward and backward stepwise procedure, with variables being entered into the model if they had a *p*-value of less than 0.05 and removed if they then had a *p*-value over 0.05 when included in the model with the other variables. The model’s steps and equations are depicted in Table 3.

The final model shows that the Asian race, foreign vaccines, adequate testing of vaccines, and the necessity of vaccine built-immunity variables are all positively correlated with booster shot support, while pharmaceutical mistrust is negatively correlated. The R square and adjusted R square values for the final model are 0.519 and 0.508, respectively.

Power estimates were generated using post-hoc analysis with G*Power 3.1. The power estimate for the logistic regression is 0.07. The power estimate for the multiple linear regression fixed model for finding any deviation from 0 is close to 1.

## 4. Discussion

Our study observed that the uptake of the COVID-19 vaccine was high among the NYITCOM medical students who responded to the survey, i.e., 95.3% (301/316). Initial studies exploring intentions of COVID-19 vaccine uptake among medical students varied by geographical locations and time—vaccine acceptance was estimated to be 23%, 35%, and 37.3% in southeast Michigan in September 2020, Egypt in January 2021, and Uganda in March 2021, respectively [44,45,46]. The low number of those abstaining from the vaccine in our study greatly impacted the power of the model to identify important variables to predict actual vaccine uptake. The low response rate of 24% (316 participants) could have impacted the types of participants in the study in many ways. Students with strong feelings about vaccination might have participated to express their attitudes, especially those who were not in favor of vaccination. Alternatively, although the survey was anonymous, social pressure from a largely vaccinated community at NYITCOM could have led those abstaining from the vaccine to be quieter about their views. Additionally, the lack of monetary incentive might not encourage those without strong opinions to respond to the survey.

Our study found that acceptance of a COVID-19 booster is a significant predictor of current COVID-19 vaccines compliance. This finding was inconsistent with the limited literature available at the time of the study. A cross-sectional study that explored individuals’ willingness to accept a yearly COVID-19 booster dose did not find booster acceptance as a significant predictor of COVID-19 vaccine uptake [47]. Medical students are more likely to be aware that vaccine-building immunity might require multiple doses to achieve a sufficient level of protection. More research is needed to explore this area of knowledge.

A vaccine mandate by schools or healthcare systems is associated with increased vaccine uptake [44]. While NYITCOM school officials did not announce a vaccine mandate at the time of the study, they encouraged students to get vaccinated and reported that COVID-19 vaccine documentation might be requested in the future, according to an announcement made by the NYITCOM Office of the President on 29 April 2021. Despite that, the school offered flexibility about requiring vaccination; vaccination exemptions due to medical or religious reasons would be evaluated on a case-by-case basis. Additionally, osteopathic medical students may perceive that the virtual or hybrid medical education offered during the pandemic was not an optimal alternative to learning human anatomy, conducting in-person clinical assessments with standardized patients, and practicing hands-on osteopathic medicine. As a result, this could have encouraged them to pursue vaccination to restore the traditional medical education that meets their medical education goals and research needs. Vaccination was also heavily encouraged in clinical years (OMS III-IV), and many clinical rotation sites are now also requiring vaccines or qualifying exceptions. The constant messages from school and student discussions among their peers about vaccination may have prompted vaccine uptake, especially since the school’s academic health center had vaccines available to its students on different occasions.

Descriptively, male gender and white race reported a higher vaccine uptake rate. However, none of these variables reached statistical significance regarding actual vaccine uptake or intentions of booster uptake except the Asian race, which was a significant predictor of intended booster uptake (t(220) = 2.194, *p* = 0.029). Our findings are consistent with the literature. Gender did not significantly predict the COVID-19 vaccine acceptance level in a cross-sectional study that explored the acceptance of the COVID-19 vaccine among medical students in Slovakia [48]. Other studies found a similar finding among university students in Czechia [49], Saudi Arabia [37], and in the northeast of Ethiopia [50]. No conclusive finding associates gender differences with a high probability of getting a flu vaccine in either the U.S. or Europe. Moreover, some studies have suggested that socio-cultural factors might influence vaccine uptake [51]. Race was not a significant predictor for vaccine uptake among medical students in southeast Michigan [44].

The Pfizer-BioNTech vaccine was the most administered vaccine among participants at 60.5% (182/316), followed by Moderna 34.2% (103/316) and J&J/Janssen 5.3% (16/316), which is consistent with the literature and current trend in the national vaccine uptake [11]. Despite the 90% effectiveness of mRNA vaccines, a study conducted to assess vaccine perception among Egyptian health care workers (HCWs) showed that 46.2% preferred the Pfizer-BioNTech vaccine over others due to long-term trust in the company and its products. This could also be related to the transparency of the information presented to the public compared to a relatively new company with no previous products in the market, Moderna [46]. The mRNA vaccines were approved in the U.S. for emergency use by the Food and Drug Administration (FDA) in December 2020, allowing them to become more available than J&J/Janssen, which was approved a few months later [7]. The Advisory Committee on Immunization Practices (ACIP) recommendations in December 2020 set the priority populations that should get vaccinated first amid the initial limited vaccine supply. Healthcare workers and residents of long-term care facilities were at the top of the list [52]. Medical students are considered part of the healthcare workforce. The FDA’s decision to pause the use of the J&J/Janssen vaccine to investigate rare cases of blood clots following vaccination might be a factor in the low vaccine uptake. These reports contributed to an increase in hesitancy to get the vaccine [53].

Our study found that mistrust in pharmaceutical companies is not a significant predictor of actual vaccine uptake (W(1) = 0.047, *p* = 0.828). Our finding was inconsistent with the literature [39,49]. A KFF COVID-19 Vaccine Monitor reported that public views about pharmaceutical motives had been lessened from 76% of the public stating that financial profit is the main motive to 58% of the public stating that pharmaceutical companies are equally interested in the common good and financial profits [16]. However, pharmaceutical trust was a significant predictor of intended booster uptake, with those saying they strongly agreed or agreed with the statement that pharmaceutical companies have prioritized financial profits over the public interest by producing the new vaccines in a short period of time being significantly less likely to support getting a booster shot (t(220) = −2.641, *p* = 0.009). This can be explained by the lack of data about long-term immunity produced by vaccines and booster dose benefit at the time of the study. Additionally, individuals might not see the booster dose as urgent as the initial vaccination and promoting boosters, at the time of the study, was more likely to be viewed as financially motivated. Moreover, our predictor of intended booster uptake might follow the same pattern reported by the KFF Vaccine Monitor.

Our analysis showed that vaccine-built immunity did not predict the actual vaccine uptake among participants (W(1) = 3.185, *p* = 0.074); this finding can be explained by the very high actual vaccine uptake of 95.2% (301/316) in our study sample and the aforementioned reasons about the lack of information about booster shot immunity at the time of the study. However, this finding was in contrast with the intended booster uptake, which was the most significant variable (t(220) = 7.566, *p* < 0.001). Students who believe in vaccine-induced immunity might view the booster dose as part of a process to build the optimal level of protection, so they might be more acceptable to an additional COVID-19 dose than others. To put this view into perspective, one shot of measles, mumps, and rubella (MMR) vaccine is 93% effective against measles, 78% effective against mumps, and 97% effective against rubella, but the second dose of MMR increases its effectiveness to 97% against measles and 88% against mumps [54]. However, if mumps outbreak occurs in places where individuals have close, prolonged contact, such as universities, an additional dose of MMR would be recommended to enhance immunity and reduce mumps-related complications [54,55].

The perceived COVID-19 risk did not significantly predict either actual vaccine uptake (W(1) = 1.543, *p* = 0.058) or booster uptake (t(220) = 1.636, *p* = 0.103), which was inconsistent with literature, as increased perceived susceptibility to COVID-19 is associated with high vaccine uptake [40]. A scoping literature review by Aw et al. identified 15 studies supporting the association of the low perception of COVID-19 risk with vaccine hesitancy. Only four studies did not find a significant correlation between perceived risk and vaccine uptake [56]. Our sample’s age could contribute to this finding, as the average age of participants was 27.1 ± 5.5 years. According to the U.S. Centers for Disease Control and Prevention (CDC), individuals 18–29 years old had the lowest hospitalization and death rates among other adult age groups [57]. Additionally, a booster dose might not be viewed as a pressing matter compared to the initial vaccination. At the time of this study, a booster shot was viewed as a supplement to fully vaccinated individuals, pending more data on how long the vaccine-induced immunity would last and who might be eligible for these additional dosages. More research is needed to confirm and explain this finding.

Vaccine-specific variables were related to adequate vaccine testing, willingness to get a vaccine manufactured outside of the U.S., and acceptance of a booster dose. None of these variables significantly predict the actual vaccine uptake, except the booster uptake (*p =* 0.08, *p* = 0.9, and *p* < 0.001, respectively). However, students who would accept non-U.S. manufactured vaccines (t(220) = 3.017, *p* = 0.003) and believed that vaccines were adequately tested (t(220) = 3.101, *p* = 0.002) were more likely to get a booster dose. The belief of the existence of a standardized process to test vaccines (initial vaccines or boosters) thoroughly before their approval might explain this finding. However, our study could not find this association with actual vaccine uptake. A previous study showed that Americans were more comfortable receiving American-manufactured vaccines than those developed outside the U.S. [47]. Views might have changed as more vaccine data have emerged from different manufacturers worldwide with high effectiveness and safety profiles, or the opinions about the vaccine’s benefits may outweigh hesitancy over foreign vaccines. Additionally, some of these vaccines have been approved by the credible, international World Health Organization [21,58].

Our study reported a promising finding of high vaccine acceptance which could mean vaccine hesitancy is a minimal issue among medical students. The WHO acknowledges that vaccine hesitancy is a complex problem that requires multi-component strategies—target unvaccinated, increase knowledge about vaccine and vaccination, tailor messages to specific subgroups, and impose vaccine mandates—to minimize vaccine refusal [21]. Gallè et al. identified a significant association between the COVID-19 vaccine acceptance and knowledge about vaccine and vaccination [19]. Schools have the opportunity to improve students’ knowledge by incorporating academic curricula that address vaccine hesitancy and provide students with the necessary resources to address the issue in the communities they serve in the future.

Our study is limited in scope, as it was conducted at one osteopathic medical school. This impacts generalizability. With the small number of those abstaining from the vaccine in this study, the potential variables explaining vaccine uptake are automatically constrained. The low response rate might have resulted in a nonresponse bias, which might mean that a lower percentage of the campus was vaccinated at the time or those who were not vaccinated chose not to share their opinions. Additionally, the lower response rate could also have eliminated the opinions of those opposed to a booster dose. This study focuses on intentions to receive the booster, but further studies should investigate actual booster uptake, as intentions do not necessarily translate into behavior. Moreover, more information is being released rapidly about vaccine-induced immunity, the benefits of a booster dose, and those who qualify for the extra shot. This means that ongoing investigations are needed to assess the attitudes around the vaccine and boosters, as these perceptions may shift as additional information is provided. While the five predictors explained over 50% of the variation in the booster variable in the model, this still means that there are some aspects of booster vaccine hesitancy that would require further evaluation.

## Figures and Tables

**Table 1:** Characteristics of study participants (N = 316).

Variable	Number (%)
**Age**	
18–19	1 (0.3%)
20–29	249 (78.8%)
30–39	33 (10.4%)
40–49	18 (5.7%)
Prefer not to answer	1 (0.3%)
**Gender**	
Female	149 (47.2%)
Male	158 (50.0%)
Other	2 (0.6%)
Prefer not to answer	5 (1.6%)
**Race/Ethnicity**	
White	182 (57.6%)
Black or African American	12 (3.8%)
American Indian or Alaskan Native	1 (0.3%)
Asian	82 (25.9%)
Native Hawaiian or other Pacific islander	0 (0%)
From multiple races	17 (5.4%)
Other	10 (3.2%)
Prefer not to answer	12 (3.8%)
**Marital status**	
Married	60 (18.9%)
Widowed	0 (0%)
Divorced	5 (1.6%)
Separated	1 (0.3%)
Never married	242 (76.6%)
Prefer not to answer	8 (2.5%)
**Household income**	
Below 10 K	98 (31.0%)
10–50 K	33 (10.4%)
51–100 K	55 (17.4%)
101–150 K	24 (7.6%)
151–200 K	10 (3.2%)
Above 200 K	18 (5.6%)
Prefer not to answer	77 (24.4%)
**Campus location**	
Jonesboro	101 (32.2%)
Old Westbury	210 (66.2%)
Prefer not to answer	5 (1.6%)
**Vaccine type**	
Pfizer-BioNTech	182/316 (60.5%)
Moderna	103/316 (34.2%)
J&J/Janssen	16/316 (5.3%)

**Table 2:** Model equation for logistic regression for vaccine uptake.

		B	S.E.	Wald	df	Sig.	Exp(B)
Step 1 ^a^	Booster	−1.211	0.344	12.408	1	<0.001	0.298
	Constant	1.011	1.175	0.740	1	0.390	2.748
Step 2 ^b^	booster	−0.955	0.330	8.359	1	0.004	0.385
	White	−28.122	500,903.041	0.000	1	1.000	0.000
	Constant	1.072	1.193	0.808	1	0.369	2.921

a. Variable(s) entered on step 1: booster. b. Variable(s) entered on step 2: White.

**Table 3:** Model equation for the linear regression of booster shot support.

Model		Unstandardized Coefficients BStd. Error		Standardized Coefficients Beta	t	Sig.
1	(Constant)	1.299	0.251		5.170	<0.001
	Build_immunity	0.712	0.056	0.646	12.666	<0.001
2	(Constant)	1.038	0.244		4.251	<0.001
	Build_immunity	0.557	0.062	0.505	9.048	<0.001
	Testing	0.252	0.050	0.282	5.053	<0.001
3	(Constant)	0.964	0.242		3.988	<0.001
	Build_immunity	0.521	0.062	0.473	8.422	<0.001
	Testing	0.204	0.052	0.229	3.938	<0.001
	Outside_US	0.123	0.044	0.153	2.796	0.006
4	(Constant)	1.526	0.330		4.622	<0.001
	Build_immunity	0.490	0.063	0.445	7.831	<0.001
	Testing	0.165	0.054	0.185	3.080	0.002
	Outside_US	0.119	0.043	0.148	2.745	0.007
	Pharma	−0.110	0.044	−0.133	−2.466	0.014
5	(Constant)	1.522	0.327		4.649	<0.001
	Build_immunity	0.473	0.062	0.429	7.566	<0.001
	Testing	0.165	0.053	0.185	3.101	0.002
	Outside_US	0.131	0.043	0.163	3.017	0.003
	Pharma	−0.117	0.044	−0.142	−2.641	0.009
	Asian	0.217	0.099	0.104	2.194	0.029

## Data Availability

The data presented in this study are available upon request from the corresponding author.
